# Biodegradable piezoelectric PHB-BT nanofiber scaffolds combined with ultrasound stimulation to accelerate bone regeneration by regulating Ca^2+^/CaN/NFAT

**DOI:** 10.7150/thno.124648

**Published:** 2026-01-22

**Authors:** Yangyang Qu, Yifeng Shang, Shixing Luo, Xiaomin Pei, Yuanming Xiao, Jinmin Zhao, Li Zheng, Chuanan Liao, Ruiming Liang

**Affiliations:** 1 Guangxi Engineering Center in Biomedical Materials for Tissue and Organ Regeneration, International Joint Laboratory on Regeneration of Bone and Soft Tissues, Guangxi Key Laboratory of Regenerative Medicine, Collaborative Innovation Centre of Regenerative Medicine and Medical Bioresource Development and Application Co-constructed by the Province and Ministry, The First Affiliated Hospital of Guangxi Medical University, Nanning 530021, China.; 2 Department of Orthopaedics Trauma and Hand Surgery, The First Affiliated Hospital of Guangxi Medical University, Nanning 530021, China.; 3 Life Sciences Institute, Guangxi Medical University, Nanning 530021, China.; 4 The Ninth Affiliated Hospital of Guangxi Medical University/Beihai People's Hospital, Beihai 536000, China.; 5 Pharmaceutical College, Guangxi Medical University, Nanning 530021, China.

**Keywords:** bone defect, piezoelectric effects, electrical stimulation, ​ osteogenesis, nanofiber scaffold

## Abstract

**Rationale:** Bone defects pose a persistent challenge in orthopedic medicine due to their limited self-repair capacity. Although guided bone regeneration scaffolds have shown therapeutic potential, their clinical efficacy remains constrained by their suboptimal osteoinductive capability.

**Methods:** Herein, we developed biodegradable piezoelectric polyhydroxybutyrate-barium titanate (PHB-BT) nanofiber scaffolds capable of generating synergistic piezoelectric stimulation for bone repair when integrated with low-intensity pulsed ultrasound (LIPUS).

**Results:** Compared with conventional PHB scaffolds, ​PHB-BT nanofiber scaffolds​ ​showed enhanced piezoelectric properties​ and ​excellent biocompatibility, ​thereby facilitating​ sustained osteogenic activity. ​*In vitro*​ studies revealed that these scaffolds ​significantly promoted​ the osteogenic differentiation of bone marrow mesenchymal stem cells under LIPUS stimulation. ​Notably, ​*in vivo*​ evaluations ​demonstrated​ that these scaffolds ​substantially accelerated bone defect repair​, with complete scaffold degradation observed after eight weeks. Mechanistically, PHB-BT nanofibers improved osteogenesis via activating the Ca^2+^/calcineurin/nuclear factor of activated T-cells signaling pathway in response to ultrasound stimulation.

**Conclusions:** These findings have significant implications for the design of next-generation, implantable electrical stimulators capable of providing sustained electromechanical cues for personalized bone tissue engineering applications.

## Introduction

A bone defect is a prevalent orthopedic condition that often arises from trauma, tumor resection, infection, or congenital genetic factors 1, 2. Various methods, including autologous and allogeneic bone transplantation and distraction osteogenesis, have been used to treat bone defects; however, achieving complete healing remains a challenging problem in orthopedics 3, 4.

By using a barrier membrane, guided bone regeneration (GBR) that promotes the formation of osteoblasts and bone proliferation offers an innovative approach for addressing bone defects 5-8. It also enhances the growth and specialization of cells derived from the same individual, thereby establishing an optimal environment for bone tissue regeneration 9. In addition to its ease of operation and avoidance of secondary surgery, GBR accelerates the repair of large segmental and irregular bone defects of tubular bone 10. However, commercially available GBR membranes, such as poly(ε-caprolactone) (PCL), collagen, and polyglycolic acid, among others, are not as effective and often result in a slow healing progression 11, which is unsuitable for clinical application. Thus, exploiting functional GBR membranes with stimulus responsiveness may broaden their application in bone regeneration.

Considering the piezoelectric nature of bones, the application of electroactive piezoelectric GBR membranes to induce bone regeneration is promising 12, 13. Piezoelectric GBR membranes may convert mechanical force into electrical stimulation (ES), and the electrical signals can further promote cell migration, proliferation, and differentiation, thus restoring the osteogenic electrical microenvironment and accelerating bone reconstruction 14, 15. Studies have demonstrated that piezoelectric signals can stimulate important cellular processes in osteoblasts or mesenchymal stem cells by activating signaling pathways, such as calcium channel, Wnt/β-catenin, and mitogen-activated protein kinase signaling 16. In particular, voltage-gated Ca^2+^ channels (VGCCs) located in cell membranes play a significant role in bone healing and regeneration 17, 18. ES increases intracellular Ca^2+^ concentrations by activating the calcineurin (CaN)/nuclear factor of activated T-cells (NFAT) pathway 19. Upon activation, NFAT undergoes dephosphorylation, leading to its translocation into the cell nucleus, thereby facilitating the regulation of osteogenic markers, such as bone alkaline phosphatase (ALP), bone morphogenic proteins (BMPs), osteopontin (OPN), osteocalcin (OCN), and runt-related transcription factor 2 (RUNX2) 20. Therefore, the design of functional piezoelectric GBR membranes that can activate the CaN/NFAT pathway to promote Ca^2+^ influx may accelerate bone healing.

Presently, a range of piezoelectric polymers, such as polyvinylidene fluoride (PVDF), poly-3-hydroxybutyrate (PHB), and poly(L-lactic acid) (PLLA), are being used for bone tissue regeneration 16, 21, 22. Among these polymers, PHB has received considerable attention because of its exceptional biocompatibility, favorable biodegradability, strong processability, and moderate mechanical properties, which render it more appealing when compared with PVDF and PLLA 23. PHB has attracted considerable attention for various medical applications, including tissue engineering, biological scaffolds, and medical implants and devices 24, 25. As a scaffold for tissue engineering, PHB can promote bone growth and improve cell communication through directly transmitting ES and electrochemical signals to cells 26. Timin *et al.* prepared a piezoelectric PHB electrospun fiber scaffold modified with bioactive composite microcapsules and found that it had excellent osteogenic properties 27. Chernozem *et al.* used calcium carbonate-mineralized PHB piezoelectric fibers as bone tissue engineering scaffolds to promote osteoblast adhesion and proliferation 28. However, the low piezoelectric coefficients and insufficient osteoinductive potential of PHB nanofibers limit their further application in bone repair. In addition, PHB-based piezoelectric composites must be activated via a complicated polarization process before interacting with cells or tissues, which is time-consuming and inconvenient for practical applications.

Low-intensity pulsed ultrasound (LIPUS) therapy, approved by the US Food and Drug Administration for bone fracture treatment, provides a noninvasive therapeutic approach 29. LIPUS subtly manipulates cellular functions by delivering delicate mechanical vibrations and stimulating the osteogenic differentiation of bone marrow stromal cells 30. More importantly, LIPUS was recently found to have a synergistic effect with piezoelectric materials in promoting bone regeneration. Chen *et al.* developed a piezodynamic therapy by combining LIPUS and a piezoelectric BaTiO_3_-coated titanium scaffold and found that this strategy could promote the osteogenic proliferation of bone marrow stromal cells 31, demonstrating the responsiveness of piezoelectric BaTiO_3_ upon LIPUS. Fan *et al.* showed that a piezoelectric BaTiO_3_-coated porous Ti_6_Al_4_V scaffold showed the best osteogenesis and osseointegration effects in the treatment of a significant segmental bone defect 32. The synergistic effect of LIPUS and piezoelectric materials on osteogenesis is attracting increasing attention. However, the combination of LIPUS and piezoelectric GBR membranes for treating bone defects has seldomly been reported.

**Scheme 1:**
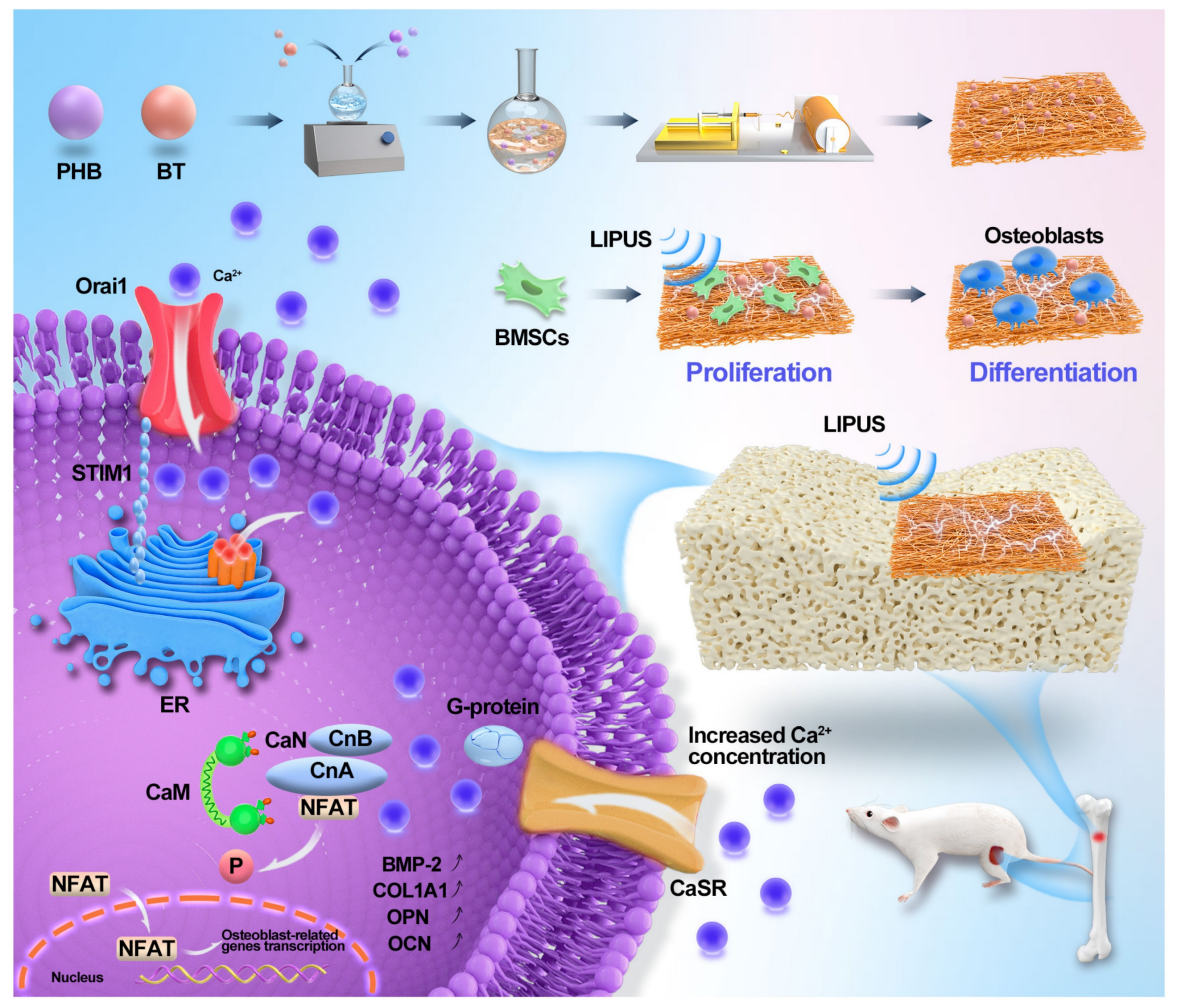
Schematic illustration of piezoelectric polyhydroxybutyrate-barium titanate (PHB-BT) nanofiber scaffolds combined with ultrasound stimulation to accelerate bone regeneration.

In this study, PHB-BT piezoelectric scaffolds were utilized as GBR membranes in combination with LIPUS to control piezoelectric charges for bone recovery (Scheme 1). Biodegradable nanofiber scaffolds of the PHB-BT piezoelectric composite were prepared with electrospinning technology and subsequently characterized. The structural morphologies, mechanical strengths, and piezoelectric properties of the scaffolds were then investigated. Synergistic therapeutic effects of the PHB-BT composite and LIPUS for treating bone defects were verified both *in vitro* and *in vivo*. Further, the mechanism underlying the osteogenic induction of PHB-BT nanofibers in combination with LIPUS stimulation was investigated. Overall, our study investigated the synergistic therapeutic potential of piezoelectric GBR nanofibers integrated with LIPUS for enhancing bone defect regeneration, building upon the evidence of improved osteogenesis through combined mechanical stimulation and ES.

## Methods

### Preparation of electrospun PHB-BT nanofibrous scaffolds

The PHB-BT nanofiber scaffolds were produced using the electrospinning technique. The electrospinning solutions were prepared by dissolving BT (Aladdin, Shanghai, China) and PHB (Aladdin) in hexafluoroisopropanol (Macklin, Shanghai, China) with varying weight ratios of 0:100, 3:97, 5:95, and 7:93 (referred to as PHB, PHB/3%BT, PHB/5%BT, and PHB/7%BT nanofiber scaffolds, respectively). The average particle size of the BT nanoparticles was 70.28 ± 14.32 nm (Figure S1), with a cubic perovskite crystal phase. The solutions were magnetically stirred for 12 h and then sonicated for 30 min. Subsequently, 5 mL of each solution was injected into a syringe. Throughout the electrospinning process, a 15.0 kV applied voltage and consistent flow rate of 0.8 mL/h were employed. The needle and copper roller, each covered with a sheet of tin foil, were kept at a constant separation distance of 15 cm. To ensure that all solvents entirely evaporated, the nanofiber scaffolds were dried in a vacuum drying oven (model LGj-10C; Foring Technology Development (Beijing) Co., Ltd., Beijing, China) after the spinning process.

### Characterization of PHB-BT nanofiber scaffolds

The structural integrity and surface morphology of PHB-BT nanofiber scaffolds were characterized through scanning electron microscopy (SEM). The electrospun scaffolds were pretreated by slicing them into 5 mm × 5 mm squares and coating with platinum through sputtering. Thereafter, the specimens were analyzed using SEM (VEGA3LMU; TESCAN, Brno, Czech Republic). The diameter distribution was determined by measuring the diameter of 50 fibers using ImageJ software (v.1.53v; National Institutes of Health, Bethesda, MD, USA). Nanostructure morphology of the PHB-BT nanofibers was examined using transmission electron microscopy (TEM) (H-7650; Hitachi, Tokyo, Japan). Crystallite structures of the PHB, BT, and PHB-BT nanofiber scaffolds were measured using X-ray diffraction (XRD) (MiniFlex600; Rigaku, Tokyo, Japan). The bonding structures of PHB, BT, and PHB-BT nanofiber scaffolds were characterized through Fourier transform infrared spectroscopy (FTIR) (Spectrum100; PerkinElmer, Waltham, MA, USA). Mechanical characterization of the electrospun PHB-BT nanofiber scaffolds was executed using a universal testing system (Instron 5943; Instron, Norwood, MA, USA) on specimens preprocessed into 20 mm × 10 mm rectangular shapes. Furthermore, the degradation test was conducted to determine durability of the piezoelectric PHB-BT nanofiber scaffolds. The experiment was initiated by measuring starting weights of the nanofiber scaffolds. Subsequently, the scaffolds were immersed in phosphate-buffered saline (PBS) and exposed to horizontal agitation at 100 rpm. The temperature was maintained at 37 °C for a period of eight weeks. The residual weight of samples (n = 3) were measured weekly after freeze-drying, and the degradation rate calculated using the following degradation formula:

Weight loss (%) = (W_0_ - W_d_)/W_0_ × 100

where W_0_ represents the initial weight of the samples and W_d_ denotes their residual weight after the experiment. An oscilloscope (ZMpoezo-B, China) was used to test the piezoelectric performance of the PHB-BT nanofiber scaffolds. Briefly, the PHB-BT nanofiber scaffolds were individually connected to the oscilloscope. The nanofiber scaffolds were then subjected to ultrasound stimulation, and the generated voltages and currents recorded by the oscilloscope. The piezoelectric constant (d_33_) of the nanofiber scaffolds was measured using a piezoelectric coefficient measuring instrument (ZILM, China). Piezoelectric performances of the PHB-BT nanofiber scaffolds were detected via piezoresponse force microscopy (PFM; Bruker, Billerica, MA, USA). Surface piezoelectric potentials of the scaffolds were also inspected using Kelvin probe force microscopy (KPFM; Bruker). Chemical analysis of the PHB-BT scaffolds was conducted using energy-dispersive X-ray spectroscopy (EDS; Bruker).

### Cell culture

Bone marrow mesenchymal stem cells (BMSCs) were extracted from newborn Sprague-Dawley (SD) rat bone marrow following ethical guidelines of the Animal Ethics Committee of Guangxi Medical University (Nanning, China). Post-isolation, the primary cell cultures were maintained under standardized culture conditions (37 ± 0.5 °C, 5% CO_2_, 95% humidity) in α-MEM medium (Biosharp, China) supplemented with 10% (v/v) fetal bovine serum (FBS) (Zhejiang Tianhang Biotechnology Ltd., Huzhou, China) and 1% penicillin-streptomycin solution (Biosharp).

### Biocompatibility of the PHB-BT nanofiber scaffolds

The PHB-BT nanofiber scaffolds were prepared in 35 mm-diameter disks and then soaked in an alcohol solution for 6 h, followed by sterilization with UV radiation for a period of 12 h. Subsequently, 1 × 10^5^ BMSCs were seeded on the PHB-BT nanofiber scaffolds in 6-well plates and incubated in an incubator at 37 °C with 5% CO_2_. Ultrasound parameters were optimized using the cell counting kit-8 (CCK-8; Dojindo, Japan) assay. The cultured BMSCs received daily LIPUS stimulation through a medical ultrasound physiotherapy device (WELLD, Shenzhen, China) with the following parameters: 1.5 MHz frequency, 0.2 ms pulse duration, 0.75 W/cm^2^ pulse strength, and 1 kHz pulse repetition frequency. Cell proliferation was evaluated on day 7 using the CCK-8 assay (n = 3), and the optical density (OD) at 450 nm measured using a microplate reader (Thermo Fisher Scientific, Waltham, MA, USA). Apoptosis of cells cultured on the PHB-BT nanofiber scaffolds (n = 3) was measured with an Annexin V-EGFP/PI apoptosis detection kit (KeyGEN BioTECH, Nanjing, China). Apoptotic cells were enumerated via flow cytometry (BD FACSCalibur; Becton Dickinson, Franklin Lakes, NJ, USA). The morphology of cells grown on the PHB-BT nanofiber scaffolds was examined via SEM. Cellular viability assessment of BMSCs was performed using a dual-fluorescence calcein-AM/PI assay (C2015L; Beyotime Biotechnology, Haimen, China) with optimized staining parameters (calcein-AM: 2 μM, PI: 5 μM), followed by high-resolution imaging on a Leica TCS SP8 confocal system (Leica, Wetzlar, Germany).

### *In vitro* osteogenic differentiation protocol

The osteogenic induction medium was formulated by combining α-MEM with 10% FBS, 10 mM β-glycerophosphate, 50 µM ascorbate, 10 nM dexamethasone, 100 µg/mL glutamine, and 1% streptomycin-penicillin. BMSCs with a 1.0 × 10^6^ cell density were co-cultured with the PHB-BT nanofiber scaffolds in 6-well plates in osteogenic medium. After a 7-day culture, calcium deposition was examined using Alizarin Red S (ARS) staining. The procedure used a 1% ARS solution (Solarbio, Beijing, China) in accordance with the manufacturer's recommendations. The images were processed with a photographic tool. Furthermore, ALP activity was measured using an ALP test kit (Beyotime Biotechnology) on days 7 and 14, with OD readings taken at 520 nm using a microplate reader.

Quantitative real-time reverse transcription PCR (qRT-PCR) was performed to investigate the osteogenic differentiation of BMSCs. Osteogenesis-related genes, including collagen type 1 (*COl1a1*), *BMP2*, ALP, *OCN*, *RUNX2*, and *OPN*, were analyzed after 7 or 14 days of culture. Total RNA was extracted from BMSCs using a HiPure Total RNA Mini Kit (Magen, Guangzhou, China), following manufacturer's protocols, whereafter cDNA synthesis was performed with the PrimeScript RT reagent Kit (Takara, Shiga, Japan). Gene expression was quantified on a LightCycler 480 real-time PCR system (Roche, Basel, Switzerland) using the Universal SYBR Green Fast qPCR Mix (ABclonal, Wuhan, China), with glyceraldehyde-3-phosphate dehydrogenase (GAPDH) as an endogenous control. Data analysis was performed using the 2^-ΔΔCt^ method normalized to GAPDH expression levels. The primers used for qRT-PCR analysis are listed in Table S1.

### Intracellular Ca^2+^ assay

Intracellular Ca^2+^ levels were assessed using a Fluo-4 AM fluorescence probe (Beyotime). In 6-well plates, 1.0 × 10^6^ BMSCs were seeded on the PHB-BT nanofiber scaffolds and subjected to daily LIPUS stimulation. Following a 7-day period of incubation, the cells were incubated in medium with 5 μM Flow-4 AM for 1 h. Thereafter, the cells were rinsed thrice with PBS. Fluorescence images were obtained using confocal laser scanning microscopy.

### Western blotting analysis

Western blotting was conducted to determine the protein expression levels of NFAT, CaN, and calmodulin (CaM) in BMSCs. Briefly, BMSCs were cultured on the PHB-BT nanofiber scaffolds under LIPUS stimulation. Cellular lysis was performed using RIPA buffer. Total protein was quantified using a BCA assay (P0012S; Beyotime) following the manufacturer's protocol.

Equal amounts (30 μg) of protein lysates were separated via sodium dodecyl sulfate-polyacrylamide gel electrophoresis (10% polyacrylamide gel, P0012AC; Beyotime) and transferred to PVDF membranes (0.45 μm; Biosharp) using a semi-dry blotting system (15 V, 60 min). Membranes were blocked with 5% (w/v) bovine serum albumin (Beyotime) in TBS-T buffer (20 mM Tris, 150 mM NaCl, 0.1% Tween-20) for 1 h at room temperature (~25°C), followed by overnight incubation with the following primary antibodies overnight at 4 °C: anti-CaM antibody (1:1000; Abcam, Cambridge, UK), anti-CaN (1:1000; Abcam), anti-NFAT-5 (1:500; Santa Cruz Biotechnology, San Diego, CA, USA), and anti-GAPDH (Thermo Fisher Scientific). The membrane was then incubated with rabbit anti-goat IgG (H+L) secondary antibody (81-1620; Invitrogen, Waltham, MA, USA). The protein bands were developed using a BeyoECL Plus assay kit (Beyotime) and recorded with an Amersham Imager 600 System (GE Healthcare, Chicago, IL, USA). The intensity of the western blotting bands in grayscale was measured using ImageJ software.

### *In vivo* bone defect repairing

The Animal Ethics and Welfare Committee of Guangxi Medical University granted ethical authorization for this study (protocol number: SCXK-Gui-2022-0015). SD rats, aged between 8 and 10 weeks and weighing 250-300 g, were sourced from the Animal Center of Guangxi Medical University. The SD rats were maintained under climate-controlled conditions, with a 25 ± 3 °C temperature and 40-60% relative humidity. The rats were provided normal food and water. Prior to the experimental procedures, the rats were rendered unconscious via intraperitoneal injection of pentobarbital sodium. A cylindrical defect (3 mm diameter × 2.5 mm depth) was drilled into the proximal end of the rat tibia using a 3.0 mm drill bit. The hind legs of SD rats were surgically fitted with PHB or PHB-BT nanofiber scaffolds, and the skin and myofascial membranes painstakingly sewn together. A total of 72 SD rats were included in the *in vivo* experiments (n = 6/group), with 36 rats assessed at each time point. Subsequently, the 72 SD rats were randomly assigned to six groups; namely the (1) control: untreated defects; (2) LIPUS group: defect treatment with LIPUS stimulation; (3) PHB group: defect treatment with PHB nanofiber scaffolds; (4) PHB+LIPUS group: defect treatment with PHB nanofiber scaffold and LIPUS stimulation twice a week for 10 min; (5) PHB/5%BT group: defect treatment with PHB/5%BT nanofiber scaffold; (6) PHB/5%BT+LIPUS group: defect treatment with PHB nanofiber scaffold and LIPUS stimulation twice a week for 10 min. The parameters of the ultrasound system included a 1.5 MHz frequency, 0.75 W/cm^2^ pulse strength, 0.2 ms pulse duration, and 1 kHz pulse repetition frequency. Following the surgical procedures, tibial samples were collected at 4 and 8 weeks and subsequently fixed with 4% paraformaldehyde for further examination.

### Histological staining

After undergoing surgical procedures for 4 or 8 weeks, the SD rats were sacrificed under isoflurane anesthesia. Subsequently, the repaired tibial specimens were harvested for gross observation and histological staining. Following visual inspection and photography, the tibia that underwent repair was immobilized in 4% paraformaldehyde for 48 h and subsequently decalcified using ethylenediaminetetraacetic acid. Histological staining was conducted using hematoxylin-eosin (HE) staining with an HE stain kit (G1120; Solarbio), and immunohistochemical staining (Col1a1) with a universal two-step immunohistochemical kit (PV-9000; ZSGB-Bio, Beijing, China). Digital images were acquired using an optical microscope (DMIL LED; Leica) for photographic documentation.

### Micro-CT analysis

Bone regeneration in the tibial defects was quantitatively assessed using micro-CT (SkyScan 127; Bruker) after 4- and 8-week treatment periods. Scanning was performed with a spatial resolution of 18 μm, X-ray tube voltage of 100 kV, and tube current of 100 mA. The MIMICS System (Materialise Co., Leuven, Belgium) was used to recreate and evaluate 3D models of the tibia. Quantitative bone parameters, including bone volume fraction (BV/TV), trabecular thickness (Tb.Th), and trabecular separation (Tb.Sp), of the samples from each group were calculated.

### Statistical analysis

The experimental data were provided as the mean value ± standard deviation. Statistical analysis was conducted using SPSS v.20.0 software (IBM, Armonk, NY, USA). The experimental data were subjected to one-way analysis of variance followed by Tukey's *t*-test. Statistical significance was defined as p < 0.05.

## Results

### Characterization of PHB-BT nanofiber scaffolds

**Figure 1:**
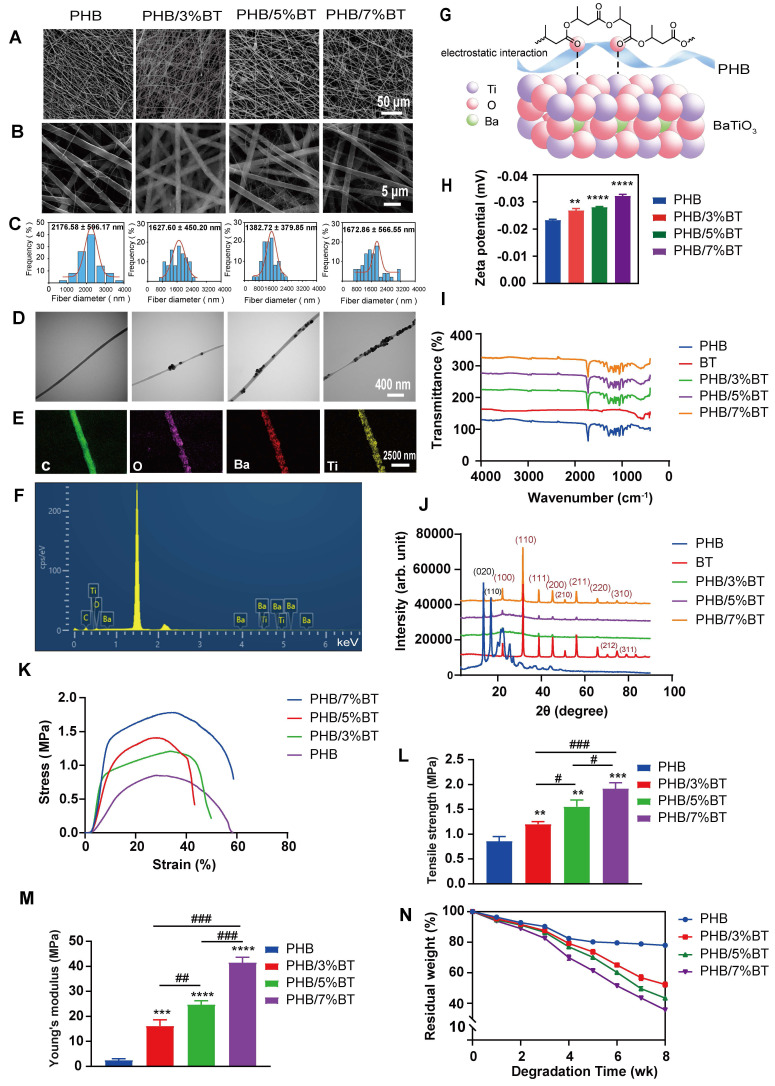
** Characterization of the polyhydroxybutyrate-barium titanate (PHB-BT) nanofiber scaffolds.** A-B: Scanning electron micrographs of the PHB and PHB-BT nanofiber scaffolds. Panels A (magnification: 1000×) and B (magnification: 8000×) show independently magnified observations of different regions. C: Diameter distribution of the PHB and PHB-BT nanofiber scaffolds. Values are expressed as mean ± standard deviation (SD) (n = 50). D: Transmission electron micrographs of the PHB and PHB-BT nanofiber scaffolds. E-F: Energy-dispersive X-ray spectroscopy analysis of the PHB/5%BT nanofiber scaffold. G: Molecular interactions between BT and polymer chains. H: Zeta potential of the PHB-BT scaffolds. I-J: Fourier transform infrared spectroscopy (I) and X-ray diffraction (J) analyses of the BT, PHB, and PHB-BT nanofiber scaffolds. K-M: Mechanical properties of PHB and PHB-BT scaffolds. N: Degradation rate of the PHB and PHB-BT nanofiber scaffolds. Experimental values are expressed as mean ± SD (n = 3). ^*^ p < 0.05, ^**^ p < 0.01, ^***^ p < 0.001, and ^****^ p < 0.0001 (vs. PHB group); ^#^ p < 0.05, ^##^ p < 0.01, and ^###^ p < 0.001 (intergroup comparisons).

The PHB-BT nanofiber scaffolds with gradient BT contents (0, 3, 5, and 7 wt.%) were fabricated via electrospinning. As observed in the SEM micrographs (Figure 1A-B), the three-dimensional hierarchical porous networks of electrospun scaffolds were formed by the randomly distributed nanofibers, and the PHB-BT nanofibers were uniform in size and relatively smooth on the surface. The size distribution of the PHB, PHB/3%BT, PHB/5%BT, and PHB/7%BT nanofibers confirmed their uniformity; they had coefficients of variation of approximately 27.76%, 27.94%, 27.75%, and 34.21%, respectively. Average diameters of the PHB, PHB/3%BT, PHB/5%BT, and PHB/7%BT nanofibers were calculated as 2176.58 ± 596.17, 1627.60 ± 450.20, 1382.72 ± 379.85, and 1672.86 ± 566.55 nm, respectively (Figure 1C). The average diameter of PHB-BT nanofibers was smaller than that of pure PHB, likely because of the increased conductivity of the electrospinning solution induced by BT. Increasing the conductivity of the electrospinning solution would lead to a high charge density in ejected jets and increase the electric force exerted on the nanofibers, resulting in decreased diameters of the electrospun nanofibers 33. Micromorphologies of the PHB-BT nanofibers and BT nanoparticles were further observed using TEM (Figure 1D). The average particle size of BT nanoparticles was 70.28 ± 14.32 nm (Figure S1). The TEM micrographs show that the BT nanoparticles were randomly dispersed on PHB-BT nanofibers, forming an irregular surface, and the intensity increased with increasing BT nanoparticle concentration. These results indicate that BT nanoparticles were successfully incorporated into the PHB-BT nanofibers.

The chemistry of the PHB/5%BT nanofiber scaffold was tested via EDS (Figure 1E-F). Corresponding EDS mapping of the C, O, Ti, and Ba elements further confirmed the uniform distribution of BaTiO_3_ nanoparticles in the PHB-BT nanofiber scaffolds. The molecular interactions between PHB and BaTiO_3_ are shown in Figure 1g. Such electrostatic interactions between Ti and dative oxygen from the carbonyl group of PHB could introduce lattice distortions. The zeta potential of PHB/3%BT, PHB/5%BT, and PHB/7%BT were -0.026, -0.028, and -0.032 mV, respectively, demonstrating a significant enhancement in piezoelectric response compared to that of pure PHB (-0.023 mV) (Figure 1H). FTIR and XRD analyses were performed to further investigate the chemical composition of the PHB-BT nanofiber scaffolds. The FTIR spectrum (Figure 1I) showed clearly defined absorption peaks. The existence of BaTiO_3_ may be inferred from the stretching and bending vibrations of Ti-O, as observed at 423 cm^-1^ and 550 cm^-1^, respectively. In addition, the peak identified at 3310 cm^-1^ indicates terminal stretching of the O-H vibrations, which are characteristic of PHB. The peaks observed at 2973, 2889, and 2741 cm^-1^ are indicative of the stretching vibrations of C-H bonds. The peak detected at 1726 cm^-1^ is attributed to the stretching vibration of C=O bonds, while the peak at 1283 cm^-1^ corresponds to the stretching vibration of C-O bonds. As shown in the XRD patterns (Figure 1J), BaTiO_3_ exhibited characteristic peaks at 22.0°, 31.4°, 38.8°, 45.2°, 50.9°, and 65.8°, corresponding to the (100), (110), (111), (200), (210), and (220) crystal planes, respectively. The BT nanoparticles were in a cubic perovskite crystal phase. The diffraction peaks at 13.6° and 16.9° are attributed to the (020) and (110) crystal planes of PHB, respectively. The main diffraction peaks of BaTiO_3_ and PHB appeared in the XRD patterns of the PHB-BT nanofiber scaffolds, confirming that BaTiO_3_ was successfully blended into the PHB nanofiber 34, 35.

Mechanical characterization of the electrospun PHB-BT nanofiber scaffolds was performed, as shown in Figure 1K-M. Basing on that of the pure PHB nanofiber scaffold, the tensile strength of PHB/3%BT, PHB/5%BT, and PHB/7%BT nanofiber scaffolds increased by 42.86%, 84.52%, and 128.57%, respectively. Meanwhile, the Young's modulus of the PHB/3%BT, PHB/5%BT, and PHB/7%BT nanofiber scaffolds increased by 554.25%, 900.81%, and 1580.16%, respectively, compared with that of the PHB nanofiber scaffold. The results revealed a significant enhancement in the mechanical properties of PHB-BT nanofiber scaffolds with the incorporation of BT nanoparticles.

The biodegradability of nanofiber scaffolds was studied by incubating the PHB and PHB/5%BT nanofiber scaffolds in PBS for eight weeks. Degradation analysis (Figure 1N) revealed that the PHB nanofiber scaffold retained 82.47% and 77.96% of its initial weight after four and eight weeks, respectively. The PHB/3%BT, PHB/5%BT, and PHB/7%BT nanofiber scaffolds retained 79.24%, 77.02%, and 69.91% of their initial weights after four weeks and 52.28%, 43.42 %, and 35.03% after eight weeks, respectively. These results indicate that the incorporation of BT nanoparticles accelerated the degradation of PHB-BT nanofiber scaffolds, with a higher BT content correlating to a faster degradation rate.

**Figure 2:**
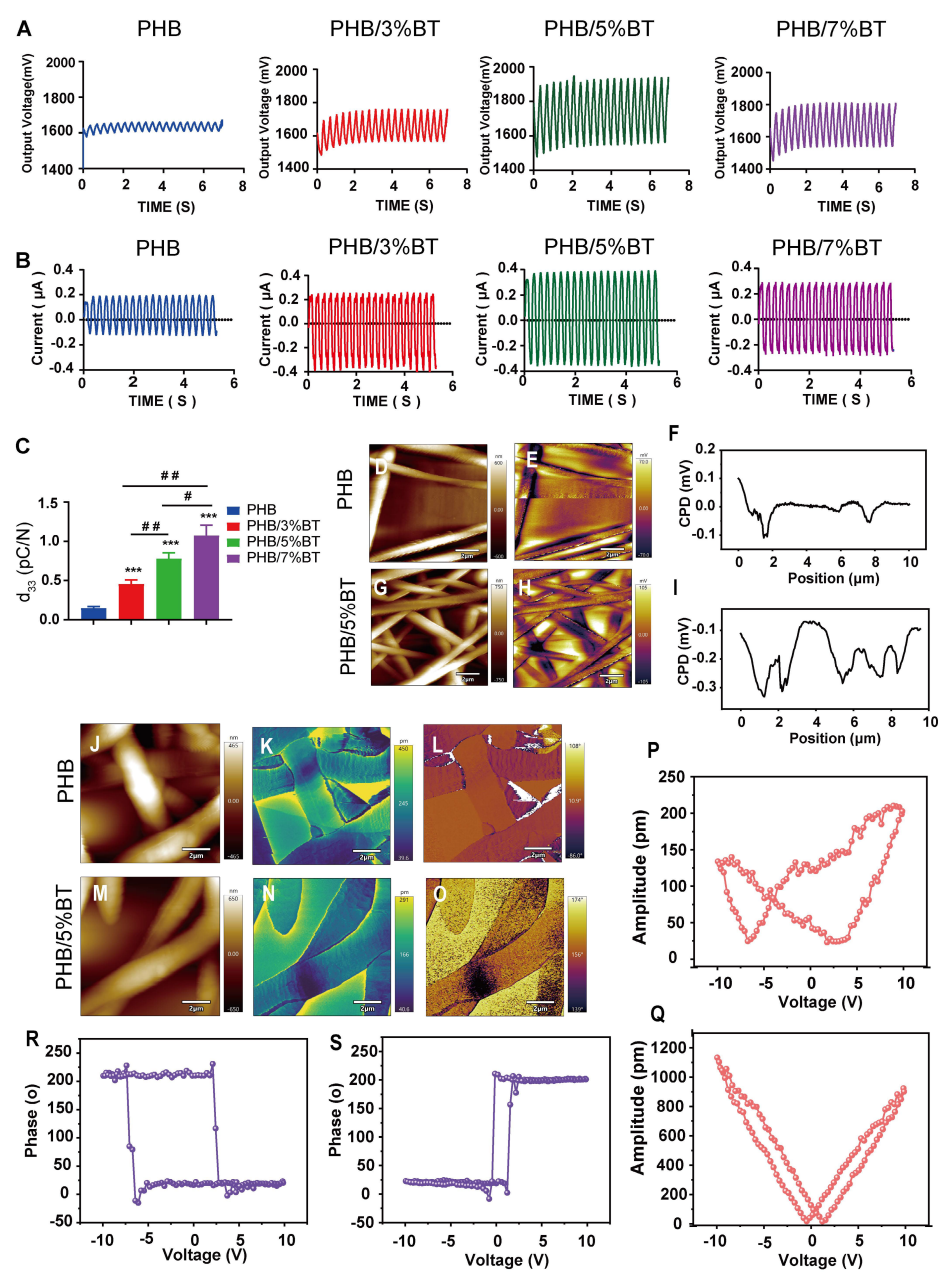
** Piezoelectric properties of the polyhydroxybutyrate-barium titanate (PHB-BT) nanofiber scaffolds**. A-C: (A) Output voltage, (B) output current, and (C) piezoelectric constant (d_33_) of the PHB, PHB/3%BT, PHB/5%BT, and PHB/7%BT nanofiber scaffolds. Values are expressed as mean ± standard deviation (n = 3). ^***^ p < 0.001 compared with the PHB group; ^#^ p < 0.05 and ^##^ p < 0.01 for comparisons between groups. D-I: Kelvin probe force microscopy (KPFM) analysis of PHB and PHB/5%BT. Surface morphologies (D-G) and corresponding KPFM potential images (E-H) of PHB and PHB/5%BT. Surface potential from the KPFM images (F-I). J-Q: Piezoresponse force microscopy (PFM) analysis of PHB and PHB/5%BT. Surface topography maps of PHB (J) and PHB/5%BT (M). PFM amplitude images of PHB (K) and PHB/5%BT (N). PFM phase images of PHB (L) and PHB/5%BT (O). Amplitude hysteresis loops of the PHB (P) and PHB/5%BT (Q) nanofiber scaffolds. Phases of the PHB (R) and PHB/5%BT (S) nanofiber scaffolds.

The piezoelectric response of the PHB-BT nanofiber scaffolds was systematically evaluated using the output voltage, output current, and d_33_ coefficient. As shown in Figure 2A, the maximal output voltage of PHB-BT was 1948.8 mV in PHB/5%BT nanofiber scaffolds, whereas that of the PHB nanofiber scaffold was only 1674.0 mV. The electrospun PHB-BT nanofiber scaffolds exhibited superior piezoelectric performance compared with that of the PHB control. The maximal output current of the PHB-BT nanofiber scaffolds was 0.395 μA in PHB/5%BT nanofiber scaffolds (Figure 2B), whereas that of the PHB nanofiber scaffolds was only 0.204 μA. The output strength of the piezoelectric signal was enhanced with increasing concentrations of BT nanoparticles, indicating that PHB-BT provides appropriate ES in response to mechanical stimulation. Finally, d_33_ coefficients of the PHB, PHB/3%BT, PHB/5%BT, and PHB/7%BT nanofiber scaffolds (Figure 2C) were 0.15, 0.457, 0.776, and 1.073 pC/N, respectively, indicating that the piezoelectric coefficients were superior to that of the PHB nanofiber scaffold with increasing incorporation of piezoelectric BT nanoparticles. The d_33_ coefficient of PHB-BT nanofiber scaffolds was comparable to that of biological bone (varies between 0.7 and 2.3 pC/N), indicating that the piezoelectric PHB-BT nanofiber scaffolds have the potential to create a biomimetic electrical microenvironment conducive to cell function and bone tissue regeneration 36.

KPFM and PFM were used to further characterize the piezoelectric properties of PHB and PHB/5%BT nanofiber scaffold surfaces. Surface piezoelectric potentials of the nanofiber scaffolds were inspected using KPFM (Figure 2D-I). The results clearly revealed that the surface potential of PHB/5%BT was higher than that of PHB (Figure 2E, H). The higher surface potential observed for PHB/5%BT (Figure 2F-I) suggests a more pronounced electrical polarization at the surface, which is a key characteristic beneficial for piezoelectric applications 37. The enhanced surface potential is expected to contribute to a stronger piezoelectric response. The piezoelectric effects of PHB and PHB/5%BT nanofiber scaffolds were investigated using PFM (Figure 2J-Q). Figure 2J and Figure 2M display the morphologies of PHB and PHB/5%BT nanofiber scaffolds. The observed amplitude-voltage (AV; butterfly loop) and phase-voltage (hysteresis loop) curves of the PHB and PHB/5%BT nanofiber scaffolds at 10 V are shown in Figure 2K-N and Figure 2L-O, respectively. The ferroelectric domain switching behavior manifests as a hysteretic loop with a 180° phase reorientation when subjected to a 10 V DC bias, demonstrating characteristic polarization switching at microstructural interfaces. The converse d₃₃ coefficient of PHB/5%BT nanofiber scaffolds was quantified as 90.22 pm/V​ through piezoelectric AV hysteresis loop analysis, exhibiting a 3.4× enhancement​ compared with that of the neat PHB (20.55 pm/V) under identical testing conditions. The piezoelectric amplitude of the PHB/5%BT nanofiber scaffold was enhanced after the incorporation of BT nanoparticles. The experimental data demonstrate that the PHB/5%BT nanofiber scaffold exhibited exceptional piezoelectric performance.

### Cell viability and cell morphology of BMSCs on PHB-BT nanofiber scaffolds with LIPUS stimulation

**Figure 3:**
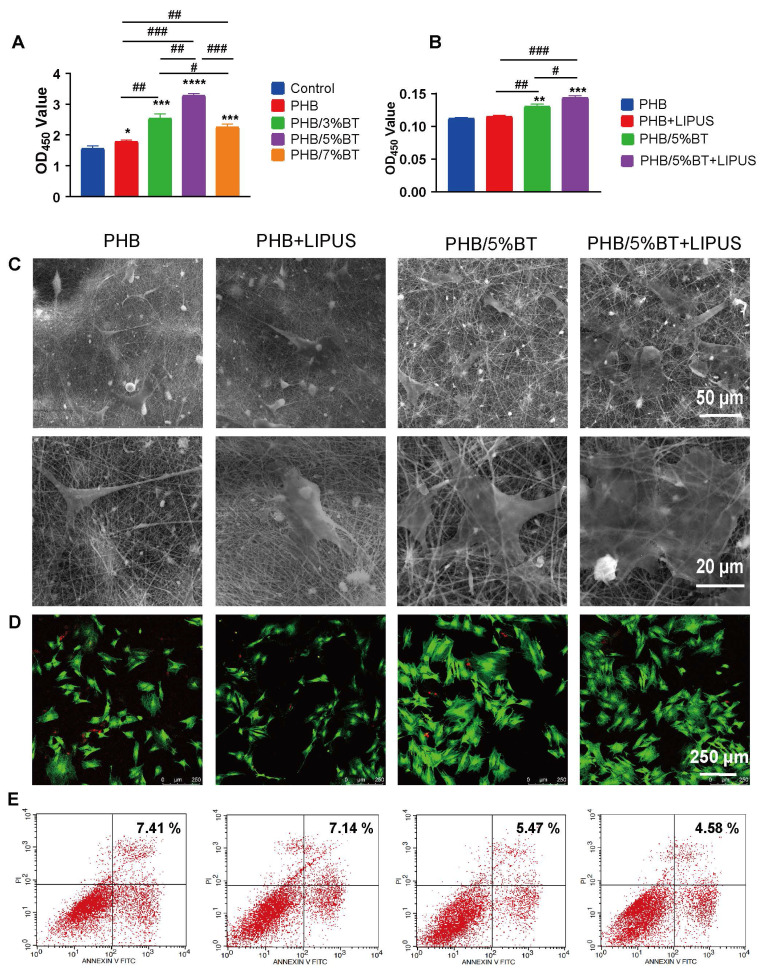
** Proliferation and apoptosis of bone marrow mesenchymal stem cells on the polyhydroxybutyrate (PHB) and PHB/5%-barium titanate (BT) nanofibers scaffolds with low-intensity pulsed ultrasound (LIPUS) stimulation.** A: Proliferation of BMSCs on PHB-BT nanofiber scaffolds. B: Proliferation of BMSCs on PHB and PHB/5%BT with or without LIPUS stimulation after seven days. C: Cell morphology of the PHB-BT nanofiber scaffolds was observed using scanning electron microscopy (magnification: 1000× and 3000×). D: BMSC viability was investigated using a calcein/PI cell viability assay. E: BMSC apoptosis was detected through flow cytometry after seven days. Experimental values are expressed as mean ± SD (n = 3). ^*^ p < 0.05, ^**^ p < 0.01, ^***^ p < 0.001, and ^****^ p < 0.0001 (vs. PHB group); ^#^ p < 0.05, ^##^ p < 0.01, and ^###^ p < 0.001 (intergroup comparisons).

The proliferation efficiency of BMSCs on the PHB-BT nanofiber scaffolds was tested over a 7-day culture period using the CCK-8 assay. As shown in Figure 3A, cell proliferation was higher on all the PHB-BT nanofiber scaffolds than on the PHB nanofiber scaffolds, demonstrating their good biocompatibility and the positive effect of BT nanoparticles on BMSC growth. In particular, BMSCs on the PHB/5%BT nanofiber scaffold exhibited the highest cell proliferation, with an increase of 109.91% compared with those on the other PHB-BT nanofiber scaffolds, confirming the superior effect on BMSC growth. Consequently, the PHB/5%BT nanofiber scaffold was selected for further experiments based on its mechanical, piezoelectric, and cell proliferation properties. The proliferation of BMSCs on PHB/5%BT nanofiber scaffolds under LIPUS stimulation was also studied after seven days. Ultrasound parameters were optimized using the CCK-8 assay (Figure S2). Compared with the control group, groups exposed to 0.35​ and 0.75 W/cm^2^​ exhibited significantly higher cell proliferation. In contrast, when the ultrasonic intensity increased to 1.00 W/cm^2^, cell proliferation declined and approached levels similar to those of the control group, implying that higher-intensity ultrasound may inhibit cell proliferation. As the exposure time was extended from 0 to 90 s, cell proliferation gradually increased. The 90 s group showed the highest value, indicating that this period​ was the optimal exposure duration for promoting cell proliferation. When the exposure time was further extended to 120 s, the OD_450_ value significantly decreased compared with that of the 90 s group, suggesting that an excessive exposure period may have a negative impact on cell proliferation. Therefore, the optimal ultrasound parameters for BMSC proliferation on the PHB/5%BT nanofiber scaffold was a 0.75 W/cm^2^ pulse strength and 90 s ultrasound time. As illustrated in Figure 3B, the proliferation of BMSCs in the PHB/5%BT and PHB/5%BT+LIPUS groups increased by 17.11% and 28.82%, respectively, compared with that in the PHB group. To demonstrate that the PHB/5%BT scaffold generated a piezoelectric electrical output under the same LIPUS parameters, the output voltage and current were tested in cell culture experiments. As shown in Figures S3 and S4, the maximal output voltage and current of the PHB/5%BT nanofiber scaffold were 824.05 mV and 0.485.13 nA, respectively, indicating that appropriate ES was generated in response to LIPUS stimulation in the biological experiments.

The morphology of BMSCs grown on the PHB/5%BT nanofiber scaffold for seven days was visualized through SEM. As demonstrated in Figure 3C, with filopodia maintaining good contact with neighboring fibers, BMSCs spread freely along the randomly oriented nanofibers. The cell intensity on the nanofiber surface of PHB/5%BT was higher than that of the PHB and PHB+LIPUS groups, whereas the PHB/5%BT+LIPUS group had the highest cell quantity. The viability of BMSCs on the PHB and PHB/5%BT nanofiber scaffolds with or without LIPUS stimulation on day 7 was further investigated through confocal microscopy using a calcein/PI assay. As shown in Figure 3D, the number of viable cells (green) on the PHB/5%BT nanofiber scaffold was greater than that of the PHB and PHB+LIPUS groups. Additionally, the PHB/5%BT+LIPUS group had more viable BMSCs than the PHB/5%BT group did. The apoptosis of BMSCs on the PHB/5%BT nanofiber scaffolds was evaluated via flow cytometry after seven days of incubation. Apoptosis rates in the PHB/5%BT (5.47%) and PHB/5%BT+LIPUS (4.58%) groups were lower than those in the PHB (7.41%) and PHB+LIPUS (7.14%) groups (Figure 3E).

### Osteogenic potential of BMSCs on PHB-BT nanofiber scaffolds under LIPUS stimulation

**Figure 4:**
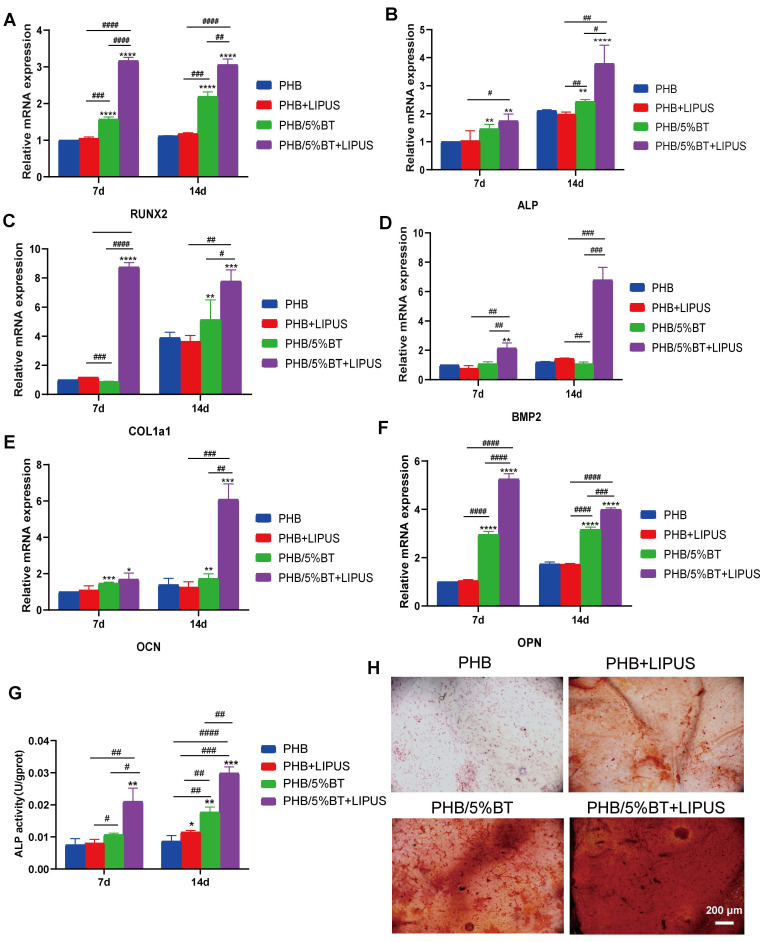
** Osteogenic differentiation of bone marrow mesenchymal stem cells (BMSCs) on polyhydroxybutyrate-barium titanate (PHB-BT) nanofiber scaffolds under low-intensity pulsed ultrasound (LIPUS) stimulation.** A-F: Quantitative real-time reverse transcription PCR analysis of the osteogenic genes, *RUNX2* (A), *ALP* (B), *COl1a1* (C), *BMP2* (D), *OCN* (E), and *OPN* (F), expressed in BMSCs on the PHB and PHB/5%BT nanofibers under LIPUS stimulation. G: Determination of the alkaline phosphatase (ALP) activity of BMSCs on PHB and PHB/5%BT nanofiber scaffolds with LIPUS stimulation for 7 and 14 days. H: Alizarin Red S staining. Values are expressed as mean ± standard deviation (n = 3). ^*^ p < 0.05, ^**^ p < 0.01, ^***^ p < 0.001, and ^****^ p < 0.0001 compared with the PHB group; ^#^ p < 0.05, ^##^ p < 0.01, ^###^ p < 0.001, and ^####^ p < 0.0001 for comparison between groups. BMP: bone morphogenic protein; COL1a1: collagen type 1; OCN: osteocalcin; OPN: osteopontin.

To investigate the osteogenic differentiation of BMSCs on PHB and PHB/5%BT nanofibers with LIPUS stimulation, the gene expression levels of osteogenic differentiation markers, including *RUNX2*,* ALP*,* COl1a1*,* BMP2, OPN*, and* OCN*, were detected using qRT-PCR on days 7 and 14. Gene expression levels between the PHB and PHB+LIPUS groups did not show notable differences (Figure 4A-F), indicating that LIPUS stimulation without piezoelectric nanofibers had little impact on the osteogenic differentiation of BMSCs. Importantly, both the PHB/5%BT and PHB/5%BT+LIPUS groups showed significantly upregulated expression of osteogenesis-related genes in BMSCs. Particularly in the PHB/5%BT+LIPUS group, the gene expression levels of *RUNX2, ALP, COl1a1, BMP2, OCN*, and* OPN* increased by 206.20%, 278.87%, 678.80%, 579.46%, 505.72%, and 299.12%, respectively, compared with those in the PHB group at day 14. The gene expression results indicated that PHB-BT nanofiber scaffolds successfully enhanced the osteogenic differentiation of BMSCs in combination with LIPUS stimulation.

The ALP activity and mineralization levels of BMSCs were examined to further evaluate the osteogenic capability of PHB-BT nanofiber scaffolds. The ALP activity in the PHB/5%BT and PHB/5%BT+LIPUS groups increased by 104.63% and 245.65%, respectively, in comparison with that in the PHB +LIPUS group, which only increased by 32.83% at day 14 (Figure 4G). The mineralization levels of BMSCs were assessed using ARS staining following a 7-day incubation period. As shown in Figure 4H, both the PHB and PHB+LIPUS groups exhibited fewer mineral deposits than the PHB/5%BT group did. In contrast, the PHB/5%BT+LIPUS group produced larger amounts of calcium deposits than the other groups did, highlighting the stronger potential of the PHB/5%BT nanofiber scaffold to facilitate mineralization under LIPUS stimulation.

**Figure 5:**
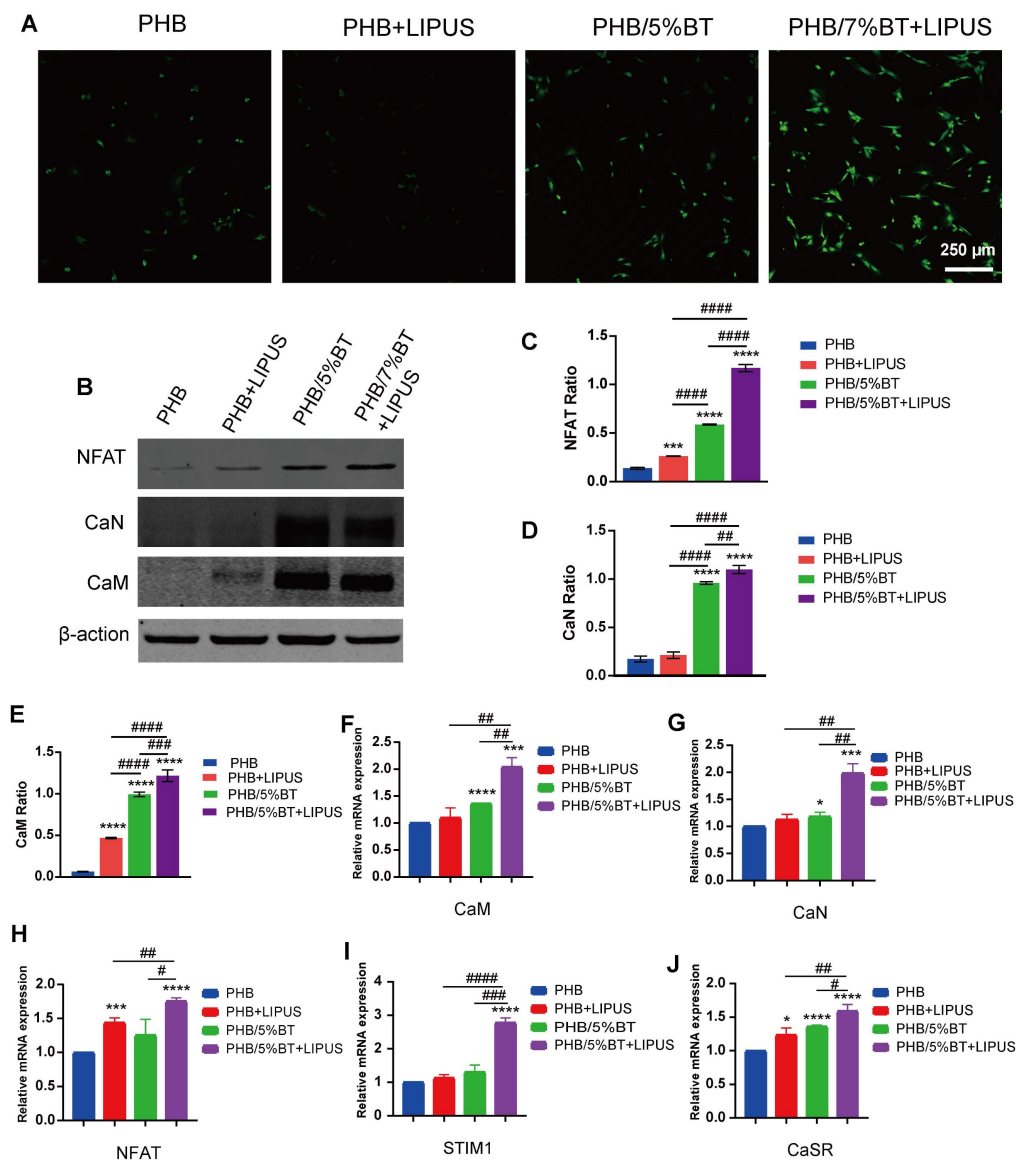
** PHB/5%BT nanofiber scaffold promoted osteogenesis with ultrasound stimulation by regulating the Ca^2+^/CaN/NFAT signaling pathway.** A: Intracellular calcium (Ca^2+^) assay. B-E: Western blotting analysis of the protein expression levels of nuclear factor of activated T-cells (NFAT), calcineurin (CaN), and calmodulin (CaM) in the Ca^2+^/CaN/NFAT signaling pathway. F-J: Quantitative real-time reverse transcription PCR analysis of the expression levels of the marker genes, *CaM*, *CaN*, *NFAT*, *STIM1*, and *CaSR* in the Ca^2+^/CaN/NFAT signaling pathway. Experimental values are expressed as mean ± standard deviation (n = 3). ^*^ p <0.05, ^**^ p < 0.01, ^***^ p < 0.001, and ^****^ p < 0.0001 (vs. PHB group); ^#^ p <0.05, ^##^ p < 0.01, ^###^ p < 0.001, and ^####^ p < 0.0001 (inter-group comparisons). LIPUS: low-intensity pulsed ultrasound; PHB-BT: polyhydroxybutyrate-barium titanate.

The Ca^2+^/CaN/NFAT signaling pathway is recognized as a vital component for facilitating osseointegration and bone production. To elucidate the mechanism underlying PHB-BT nanofiber scaffold-mediated osteogenesis, intracellular Ca^2+^ levels and the expression levels of genes and proteins in the Ca^2+^/CaN/NFAT signaling pathway were determined. The intracellular Ca^2+^ levels were measured using a Fluo-4 AM fluorescent probe and visualized via confocal microscopy. As illustrated in Figure 5A, intracellular Ca^2+^ levels of BMSCs in the PHB/5%BT+LIPUS group were more concentrated than those in any other group, indicating that the PHB/5%BT nanofiber scaffold with LIPUS stimulation significantly enhanced intracellular Ca^2+^ concentrations. Expression levels of the proteins related to the Ca^2+^/CaN/NFAT signaling pathway were assessed using western blotting. As illustrated in Figure 5B-E, the protein expression levels of CaN, NFAT, and CaM in the PHB/5%BT group were significantly higher than those in the PHB and PHB +LIPUS nanofiber scaffold groups. In particular, their expression levels in the PHB/5%BT+LIPUS group increased by 109.83%, 116.91%, and 121.82%, respectively, compared with those in the PHB group. Additionally, crucial gene expression levels of the Ca^2+^/CaN/NFAT signaling pathway, including *CaM*, *CaN*,* NFAT*,* STIM1* and* CaSR*, were investigated through qRT-PCR analysis. As shown in Figure 5F-J, BMSCs in the PHB/5%BT+LIPUS group exhibited the highest expression levels of *CaM*, *CaN*,* NFAT*,* STIM1*, and* CaSR*, exhibiting increases of 104.20%, 98.66%, 75.37%, 178.52%, and 59.30%, respectively, compared with those in the PHB group. Therefore, the results indicate that the LIPUS-stimulated PHB/5%BT nanofiber scaffold promoted osteogenesis by regulating the Ca^2+^/CaN/NFAT signaling pathway.

**Figure 6:**
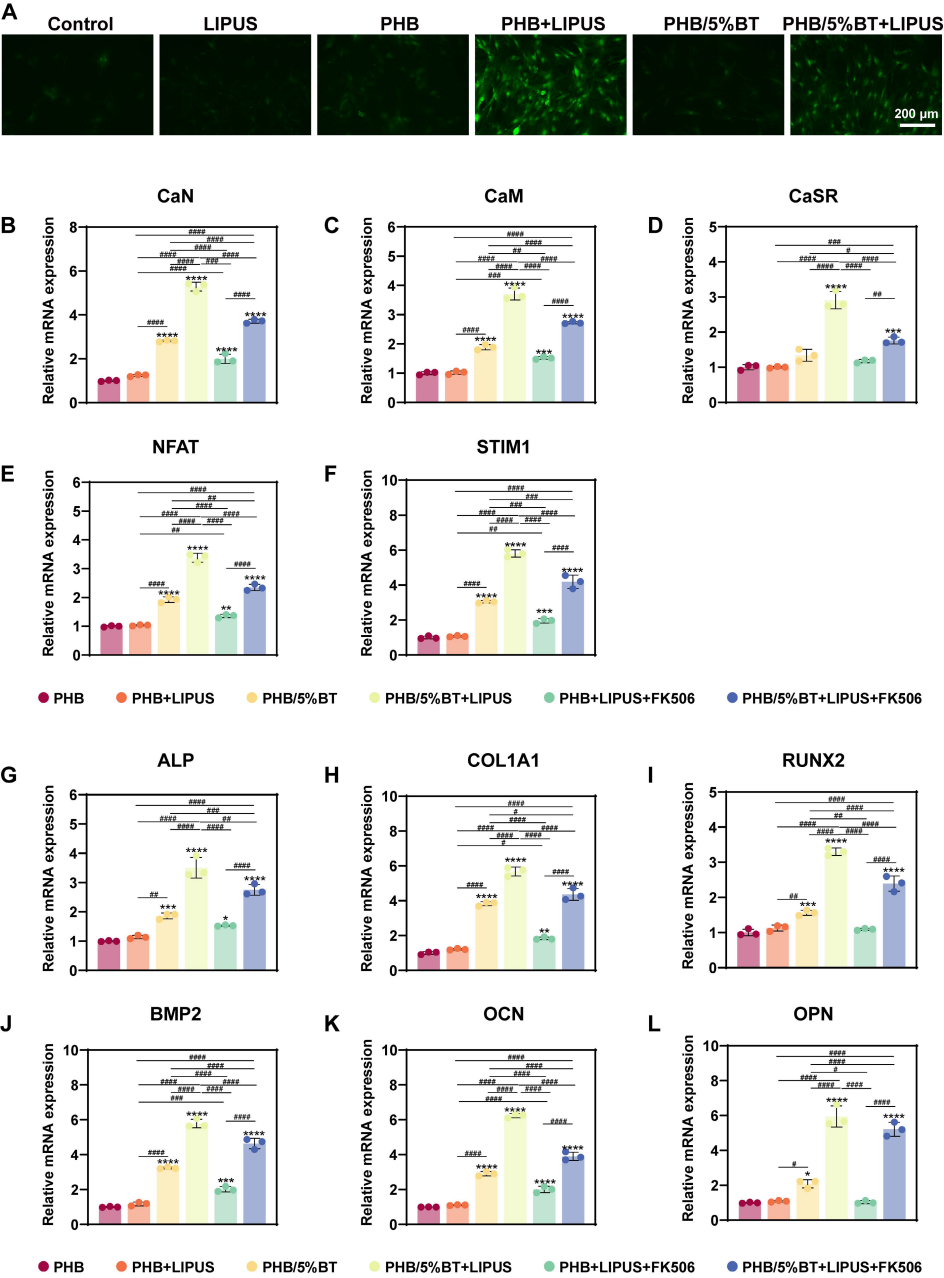
**Pharmacological and functional inhibition of the Ca²⁺/CaN/NFAT axis in regulating osteogenic differentiation.** A: Ca^2+^ ion levels in bone marrow mesenchymal stem cells (BMSCs) treated with the Ca^2+^ channel blocker, nifedipine. B-F: Quantitative real-time reverse transcription PCR (qRT-PCR) examination of the gene expression levels of the Ca^2+^/calcineurin (CaN)/nuclear factor of activated T-cells (NFAT) signaling pathway treated with the CaN inhibitor, FK506. J-L: qRT-PCR examination of the expression levels of osteogenic genes treated with the CaN inhibitor, FK506. Experimental values are expressed as mean ± standard deviation (n = 3). ^*^ p <0.05, ^**^ p <0.01, ^***^ p <0.001, and ^****^ p <0.0001 (vs. PHB group); ^#^ p <0.05, ^##^ p <0.01, ^###^ p <0.001, and ^####^ p <0.0001 (inter-group comparisons). ALP: alanine phosphatase; BMP: bone morphogenic protein; CaM: calmodulin; COL1A1: collagen type 1; LIPUS: low-intensity pulsed ultrasound; OCN: osteocalcin; OPN: osteopontin; PHB-BT: polyhydroxybutyrate-barium titanate; RUNX2: runt-related transcription factor 2.

Several studies have shown that piezoelectric scaffolds can generate a weak current to activate Ca^2+^ ion channels and enhance the osteogenic differentiation of BMSCs 38, 39. To elucidate the regulatory mechanisms underlying osteogenic differentiation, pharmacological inhibition was employed to demonstrate whether the Ca²⁺/CaN/NFAT axis serves as the primary regulator of osteogenic differentiation. Nifedipine is an L-type VGCC blocker. After treatment with nifedipine, the Ca^2+^ ion levels of BMSCs significantly reduced (Figure 6A), indicating that the piezoelectric PHB/5%BT scaffold could activate Ca^2+^ ion channels. A functional inhibition experiment (using FK506 to inhibit CaN) was performed to further investigate this mechanism. Compared with the PHB/5%BT+LIPUS group, the expression levels of CaN, CaM, NFAT, STIM1, and CaSR decreased by 29.79%, 26.38%, 30.58%, 27.97%, and 39.53%, respectively, after treatment with FK506 in the PHB/5%BT+LIPUS group (Figure 6B-F).

Meanwhile, the gene expression levels of osteogenic differentiation markers, including *RUNX2*,* ALP*,* COl1a1*,* OPN*,* OCN*, and* BMP2*, were detected via qRT-PCR on day 14. As shown in Figure 6G-L, the expression of osteogenesis-related genes in BMSCs was significantly upregulated in both the PHB/5%BT and PHB/5%BT+LIPUS groups. However, the expression levels of *RUNX2*,* ALP*,* COl1a1*,* BMP2*,* OCN*, and* OPN* decreased by 27.49%, 21.52%, 23.29%, 19.85%, 37.31%, and 12.33%, respectively, after treatment with FK506 in the PHB/5%BT+LIPUS group. These results confirm that the Ca²⁺/CaN/NFAT signaling pathway plays a critical role in the osteogenic differentiation of BMSCs on PHB/5%BT nanofibers with LIPUS stimulation.

### *In vivo* bone defect repairing

**Figure 7:**
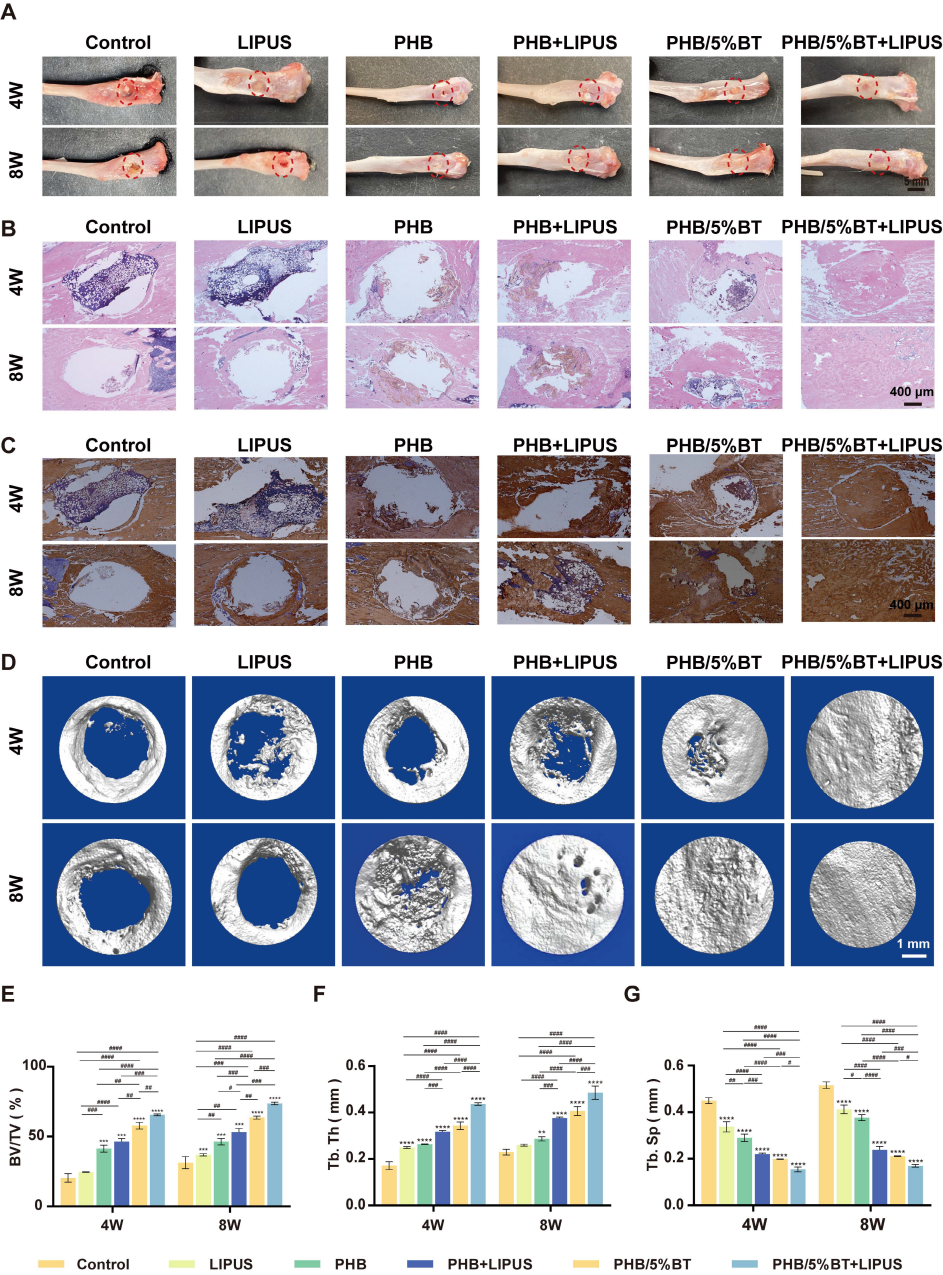
** Evaluation of the *in vivo* bone regeneration of polyhydroxybutyrate (PHB) and PHB/5% barium titanate (BT) nanofiber scaffolds with low-intensity pulsed ultrasound (LIPUS) stimulation for four and eight weeks.** A: Gross observation of tibia after treatment. B: Hematoxylin and eosin staining. C: Immunohistochemistry (Col1a1). D: Micro-CT examination. E: Bone volume/total volume (BV/TV) ratio based on the micro-CT results. F: Trabecular thickness (Tb.Th). G: Trabecular separation (Tb.Sp). Values are expressed as mean ± standard deviation (n = 3). ^*^ p <0.05, ^**^ p <0.01, ^***^ p <0.001, and ^****^ p <0.0001 compared with control group; ^#^ p <0.05, ^##^ p <0.01, ^###^ p <0.001, and ^####^ p <0.0001 for comparison between groups.

Bone regeneration capacities of the PHB and PHB-BT nanofiber scaffolds were evaluated *in vivo* using rat tibial defect models. The PHB and PHB-BT nanofiber scaffolds were implanted into freshly formed tibial defects and stimulated using LIPUS. Bone regeneration in the tibial defects was evaluated at 4 and 8 weeks after implantation. As shown in Figure 7A, no obvious signs of inflammation or necrosis were observed in the tibial specimens. Moreover, defects in the PHB/5%BT+LIPUS group were completely filled with consecutive new bone, and those in the PHB/5%BT group were almost completely healed, whereas a partially unhealed area in defects of the PHB and PHB+LIPUS groups were observed. In comparison, defects in the LIPUS and control groups remained unchanged.

Bone regeneration was further assessed using HE staining and immunohistochemistry methods. HE staining revealed that the PHB/5%BT+LIPUS group exhibited a larger amount of newly formed trabecular bone after a 4-week period than that of the other groups (Figure 7B). After eight weeks, the defect sites in the control group remained mostly unrecovered, whereas only a minimal amount of regenerated bone tissue in the LIPUS group was observed, indicating that pure LIPUS irradiation had a limited effect on osteogenesis. Moreover, histological analysis revealed that the PHB group exhibited limited osteogenic capacity, with no more than half of the defect area repaired, whereas in the PHB+LIPUS and PHB/5%BT groups, the original trabecular bone occupied more than half of the defect area. Notably, the new bone trabeculae covered nearly the entire defect site in the PHB/5%BT+LIPUS group. Col1a1 levels were detected using immunohistochemistry, and the results are presented in Figure 7C. Consistent with the HE staining observations, the immunohistochemical analysis demonstrated that the PHB/5%BT+LIPUS group exhibited the highest immunoreactivity for osteogenic markers and collagen deposition.

The osteogenic capacity of the piezoelectric PHB/5%BT nanofiber scaffold was confirmed using micro-CT evaluation. Micro-CT imaging (Figure 7D) demonstrated that the synergy between LIPUS stimulation and the PHB/5%BT nanofiber scaffold resulted in optimal tissue repair, with new bone completely filling the defect site. The new BV/TV (Figure 7E) revealed that the control, LIPUS, and PHB groups demonstrated unsatisfactory rates and quality of bone regeneration, with BV/TV ratios below 36.90 ± 0.78%, 46.27 ± 2.30%, and 53.09 ± 2.70%, respectively. The PHB/5%BT group demonstrated a more effective bone defect healing effect, as evidenced by a BV/TV ratio of 57.75 ± 2.35%. When external LIPUS stimulation was applied, the PHB/5%BT+LIPUS group showed the largest BV/TV value of 73.69 ± 0.92%. To assess the bone regeneration quality, quantitative morphometric parameters, including the Tb.Th (Figure 7F) and Tb.Sp (Figure 7G) of samples from each group, were also calculated. The PHB/5%BT+LIPUS group exhibited the most optimal synergistic therapeutic effect, with a higher Tb.Th and lower Tb.Sp than those of the other groups, indicating a denser new bone structure and greater potential for promoting bone regeneration. The results showed that the PHB/5%BT+LIPUS group exhibited a remarkable impact on bone defect repair and regeneration, primarily due to ES induced by the nanofibrous scaffold during LIPUS treatment. Furthermore, histopathological evaluation of major organs (heart, liver, lungs, and kidneys) at 8 weeks post-implantation revealed no significant abnormalities in tissue architecture or inflammatory infiltrates (Figure S5), thereby confirming the biocompatibility of PHB/5%BT scaffolds.

## Discussion

Biodegradable nanofiber scaffolds that integrate osteogenic potential and piezoelectric responsiveness represent a paradigm shift in bone regenerative medicine, offering dual-functional platforms that synergistically enhance osteogenesis. Although guided bone regeneration scaffolds have shown therapeutic potential, their clinical efficacy remains constrained by their suboptimal osteoinductive capability. Using BT nanoparticles as an osteogenic factor and piezoelectric enhancer, PHB-BT nanofiber scaffolds were successfully prepared via electrospinning and exhibited excellent piezoelectric properties, flexibility, biocompatibility, and degradability. Integration of the PHB-BT nanofiber scaffolds with LIPUS stimulation synergistically enhanced the osteogenic differentiation of BMSCs for bone repair. Mechanistically, the PHB-BT nanofibers improved osteogenesis via activating the Ca^2+^/CaN/NFAT signaling pathway in response to piezoelectric stimulation. The nanostructured piezoelectric PHB-BT nanofiber scaffolds demonstrate notable potential as biocompatible, self-powered ES platforms for implantable electronic devices, temporary medical equipment, and tissue regeneration.

The PHB-BT nanofiber scaffolds innovatively integrated biocompatibility, enhanced intrinsic piezoelectric responsiveness, and balanced mechanical properties and controllable biodegradability for bone defect repair. GBR faces challenges that extend beyond a single dimension, necessitating multifunctional membranes with mechanical stability, appropriate degradation, bioactive properties, and osteogenic induction in complex clinical scenarios 40. The design rationale of the PHB-BT nanofiber scaffold was centered on the inherent piezoelectric effect of bone tissue, as well as the excellent piezoelectric performance and proven osteogenesis of BT nanoparticles. Incorporating BT nanoparticles into PHB-BT nanofiber scaffolds can greatly enhance their mechanical and piezoelectric characteristics, resulting in an increased electrical conversion capability. As evidenced by the FITR and XRD results, BT nanoparticles were successfully doped into the PHB matrix (Figure 1I-J), which significantly improved the mechanical strength and biodegradability when compared with those of pure PHB nanofiber films (Figure 1K-N). The PHB-BT nanofiber scaffolds demonstrated the highest tensile strength of 1.92 MPa, which was significantly improved compared with that of the piezoelectric PVDF-based nanofibrous scaffolds, PVDF (0.64 MPa) and PVDF/LM-ZnO (0.93 MPa) 41, and the PCL/hydroxyapatite/PLLA-based scaffolds, Hybrid/R (1.06 MPa) and Hybrid/A (1.31 MPa) 42. Collagen membranes commonly used in clinical GBR have good biocompatibility, but they are limited by insufficient mechanical strength, unstable barrier function, fast degradation rate, and complex preparation processes 43. Compared with nondegradable metal films (such as titanium nets) and synthetic polymer membranes (such as polytetrafluoroethylene), the PHB-BT nanofiber scaffolds exhibited controllable degradation rates and sufficient mechanical strength, which are critical for maintaining a stable spatial microenvironment to support successful bone tissue regeneration. Their biodegradability eliminates the need for a second surgical procedure, and their notable bioactivity and regenerative potential further enhance their applicability in bone defect repair. The output voltage of the PHB-BT nanofiber scaffolds significantly increased from 1674.0 mV to 1948.8 mV with an increasing BT nanoparticle mass ratio (0-5%), which was higher than those of the Ph-TM nanofiber (500 mV) 44, DAT/KS (890 mV) 45, and PLLA-30-PF nanofiber membranes (1300 mV) 46. The reported d_33_ coefficient of biological bone varies between 0.7 and 2.3 pC/N, owing to the abundant presence of type I collagen fibers in its extracellular matrix (ECM) components 47. The PHB-BT nanofiber scaffolds possessed tunable piezoelectric performance, and the d_33_ coefficients of PHB/5%BT and PHB/7%BT scaffolds were 0.776 ± 0.021​ and 1.073 ± 0.034 pC/N, respectively, aligning closely with that of natural bone tissue. These synergistic features collectively position the scaffold as an ideal mechanoresponsive smart biomaterial for bone regeneration.

Electrospun nanofibers with a high specific surface area and interconnected porous structure can mimic the physical microenvironment of the ECM to provide favorable attachment sites and growth space for cells, and they have thus attracted much attention for bone regeneration applications 48. The incorporation of BT nanoparticles into PHB-BT nanofibers not only effectively improved the mechanical strength, porosity, and degradability of the scaffolds but also promoted the cell adhesion, spreading, and osteogenic differentiation of BMSCs. Compared with PHB, the PHB-BT nanofibers exhibited a notable ability to promote cell proliferation and osteogenesis, as evidenced by the CCK-8, ALP enzyme activity, qRT-PCR, flow cytometry, and ARS staining assay results (Figures 3 and 4). ES has demonstrated beneficial effects in the treatment of bone defects, notably accelerating fracture healing rates and augmenting the efficacy of bone graft procedures 49. The PHB-BT nanofiber scaffolds exhibited excellent piezoelectric properties and could generate a physiological electrical microenvironment in response to LIPUS stimulation, thereby promoting cell proliferation and differentiation. Combination of the PHB-BT nanofiber scaffolds with LIPUS stimulation enhanced the osteogenic differentiation of BMSCs by upregulating osteogenic genes and increasing ALP enzyme activity. Piezoelectric PHB-BT nanofiber scaffolds orchestrated the cellular functions of BMSCs through the synergistic interplay between physical cues (bionic structure and mechanical capacity) and electrochemical stimuli (piezoelectric potential generation), thereby establishing a multifunctional platform for advancing bone tissue engineering.

Engineered implantable PHB-BT nanofiber scaffolds were developed as multifunctional biodegradable GBR membranes and coupled with ultrasound therapy to offer a noninvasive strategy for enhancing bone defect regeneration through synergistic mechanical-electrical stimulation. Traditional ES requires cumbersome equipment, resulting in reduced patient comfort and making it difficult to provide personalized treatment, which severely hinders its clinical application 50. Consistent with the *in vitro* osteogenic potential, *in situ*-validated bone restoration was also achieved in SD rat tibial defects at 8 weeks post-implantation. Contrastingly, the PHB-BT nanofiber scaffolds exhibited excellent mechanical properties that prevent soft tissue invasion of the bone defect sites. Moreover, their superior piezoelectric performance and inherent biocompatibility synergized with deep tissue penetration and mechanical vibrations by leveraging the excellent tissue-penetrating ability of ultrasound to deliver sufficient noninvasive ES at bone defect sites, thereby achieving enhanced bone regeneration outcomes. Radiological (micro-CT and radiographic analyses) and histological (HE staining and immunohistochemistry) evaluations demonstrated that the scaffolds markedly promoted osteogenesis, as evidenced by the enhanced bone mineralization and accelerated defect healing observed. The degradation of BT may generate Ba^2+^ and TiO_2_. The degradation product, Ba^2+^, can be excreted through the kidneys or bound to plasma proteins, and some can be involved in bone mineralization, whereas TiO_2_ may be phagocytosed by macrophages and then decomposed through the lysosomal pathway 51. After treatment for eight weeks, no histopathological abnormalities or lesions were observed in the vital organs of experimental animals, indicating a favorable safety performance. Overall, the piezoelectric PHB-BT nanofiber scaffolds wirelessly transmitted mechanical energy through ultrasound, converting it into a stable electrical output and providing new insights for noninvasive and stable ES in the field of bone repair.

Given the variations in piezoelectric properties among different piezoelectric materials and their resultant differences in promoting individualized bone regeneration, the ultrasound parameters are important considerations when combining ultrasound therapy 52. Ultrasound intensity is a critical parameter that affects the activation efficiency of piezoelectric materials, which directly determines the density of microcurrents generated by the materials and the intensity of cellular responses 53. Additionally, the duration of ultrasound exposure must balance the sustained activation of piezoelectric materials with tissue tolerance 54. Wu *et al.* developed piezoelectric composite DAT/KS membranes to promote bone regeneration under ultrasound treatment (1.0 MHz, 0.3 W/cm^2^, 10 ms pulse duration, 30 min) 45. Zhao *et al.* developed a piezoelectric periosteum-bone-mimicking bilayer scaffold with ultrasound stimulation (1 MHz, 0.15 W/cm^2^, 20 min) for critical-size bone regeneration 55. Recent investigations have predominantly employed singular ultrasonic parameters (e.g., frequency and intensity) to assess bone regeneration efficacy. However, our systematic evaluation revealed that synergistic parameter combinations can significantly enhance the intrinsic piezoelectric response of PHB/5%BT nanofiber scaffolds. Through experiments analyzing ultrasonic intensity gradients (0.35-1.0 W/cm^2^) and exposure durations (0-90 s), we identified an optimized protocol (0.75 W/cm^2^, 90 s) that achieves maximal osteogenic stimulation while maintaining favorable biocompatibility. Therefore, the optimized ultrasound parameters (0.75 W/cm^2^, 90 s) were selected for further study. The BMSCs cultured on PHB/5%BT nanofiber scaffolds exhibited the highest cell proliferation under these ultrasound parameters. PHB/5%BT combined with ultrasound stimulation significantly promoted cell adhesion, osteogenic differentiation of BMSCs, and bone regeneration. We systematically evaluated the synergistic effect of PHB-BT nanofiber scaffolds with optimized ultrasound therapy on cell proliferation, osteogenic differentiation of BMSCs, and bone regeneration, thereby providing an effective alternative strategy for the clinical treatment of bone defects.

The molecular mechanism of piezoelectric materials is a key driver orchestrating multiple physiological processes for tissue repair and enhancing bone regeneration. The experimental results showed that PHB-BT nanofiber scaffolds combined with LIPUS stimulation markedly promoted osteogenesis by increasing intracellular Ca^2+^ levels. Ca^2+^ serves as a vital secondary messenger in intracellular signal transduction and is pivotal for regulating cell proliferation and osteogenic differentiation 56, 57. ES elevates intracellular Ca²⁺ concentrations through VGCCs, thereby initiating the CaM/CaN signaling cascade that mediates NFAT dephosphorylation during osteogenic differentiation 58. This process cooperatively regulates the gene expression of transcription factors and osteogenic markers, including BMPs, ALP, RUNX2, OPN, and OCN 59. In our study, the expression of CaN, NFAT, and CaM was upregulated in BMSCs cultured on PHB/5%BT under LIPUS stimulation, indicating that piezoelectric PHB-BT nanofiber scaffolds promoted the osteogenesis of BMSCs by regulating the Ca^2+^/CaN/NFAT signaling pathway (Figure 5). Cellular mechanical transduction, which converts mechanical signals into biochemical signals, is a key regulatory factor that critically involves Ca^2+^ ion signaling 60, 61. Piezoelectric PHB-BT nanofiber scaffolds can spontaneously generate charges/potentials under external ultrasonic stimulation. ES stimulates VGCCs to induce spatiotemporal calcium dynamics, characterized by extracellular Ca^2+^ influx through L-type channels and subsequent intracellular Ca^2+^ release from calcium pools 62. As a secondary messenger, calcium is involved in multiple signal transduction pathways, such as the Ca^2+^/CaN/NFAT pathway, which are related to osteogenic differentiation 20. In our study, intracellular Ca^2+^ levels in the PHB/5%BT+LIPUS group significantly improved compared with those in the other groups (Figure 6A). After treatment with the Ca^2+^ ion channel inhibitor, nifedipine, the intracellular Ca^2+^ ion levels in BMSCs significantly reduced. Functional inhibition experiments (using FK506 to inhibit CaN) also confirmed that the Ca^2+^/CaN/NFAT signaling pathway plays a crucial role in osteogenic differentiation (Figure 6). This study innovatively revealed that PHB-BT piezoelectric nanofiber scaffolds promote the osteogenic differentiation of BMSCs by activating the Ca^2+^/CaN/NFAT signaling pathway, providing critical insights for the development of next-generation implantable ES systems integrated with LIPUS for enhancing bone regeneration.

## Conclusions

In summary, piezoelectric PHB-BT nanofiber scaffolds for facilitating bone regeneration were synthesized via electrospinning. PHB-BT composite scaffolds demonstrated a significant enhancement in piezoelectric output and mechanical properties compared with those of pure PHB, along with desirable biodegradability. Combined with LIPUS stimulation therapy, PHB-BT nanofiber scaffolds significantly enhanced the osteogenic differentiation of BMSCs and repaired bone defects. Consequently, these innovative piezoelectric PHB-BT nanofiber scaffolds have the potential to be developed as biodegradable electrical stimulators for bone defect treatment.

## Supplementary Material

Supplementary figures and table.

## Abbreviations

ALP: alkaline phosphatase
ARS: Alizarin Red S
AV: amplitude-voltage
BMP: bone morphogenic protein
BMSC: bone marrow mesenchymal stem cell
BV/TV: bone volume fraction
CaN: calcineurin
CCK-8: cell counting kit-8
COl1a1: collagen type 1
d_33_: piezoelectric constant
ECM: extracellular matrix
EDS: energy-dispersive X-ray spectroscopy
ES: electrical stimulation
FBS: fetal bovine serum
FTIR: Fourier transform infrared spectroscopy
GAPDH: glyceraldehyde-3-phosphate dehydrogenase
GBR: guided bone regeneration
HE: hematoxylin-eosin
KPFM: Kelvin probe force microscopy
LIPUS: low-intensity pulsed ultrasound
NFAT: nuclear factor of activated T-cells
OCN: osteocalcin
OD: optical density
OPN: osteopontin
PBS: phosphate-buffered saline
PCL: poly(ε-caprolactone)
PFM: piezoresponse force microscopy
PHB-BT: polyhydroxybutyrate-barium titanate
PLLA: poly(L-lactic acid)
PVDF: polyvinylidene fluoride
qRT-PCR: quantitative real-time reverse transcription PCR
RUNX2: runt-related transcription factor 2
SD: Sprague-Dawley
SEM: scanning electron microscopy
Tb. Sp: trabecular separation
Tb.Th: trabecular thickness
TEM: transmission electron microscopy
VGCC: voltage-gated calcium channel
XRD: X-ray diffraction
