# Supplementary material for: Biodegradable piezoelectric PHB-BT nanofiber scaffolds combined with ultrasound stimulation to accelerate bone regeneration by regulating Ca2+/CaN/NFAT
Source: Theranostics. 2026 Jan 22;16(8):4283–303. doi: 10.7150/thno.124648 (PMC12905827; doi:10.7150/thno.124648)
Supplement: Supplementary file 1 — Supplementary figures and table. [file thnov16p4283s1.pdf]

Supplemental Material

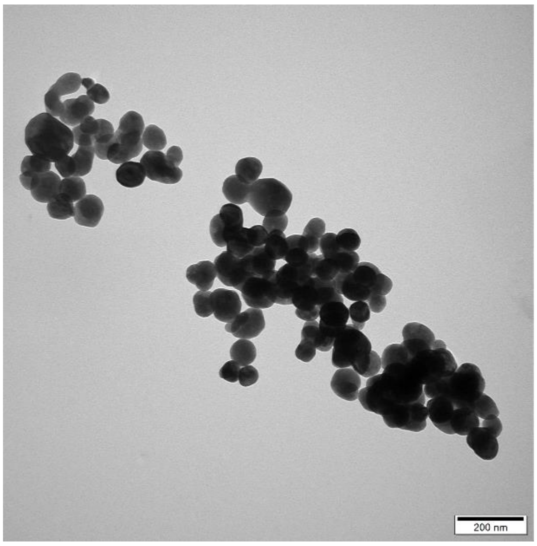

Supplementary Figure S1. Transmission electron micrographs of barium titanate nanoparticles.

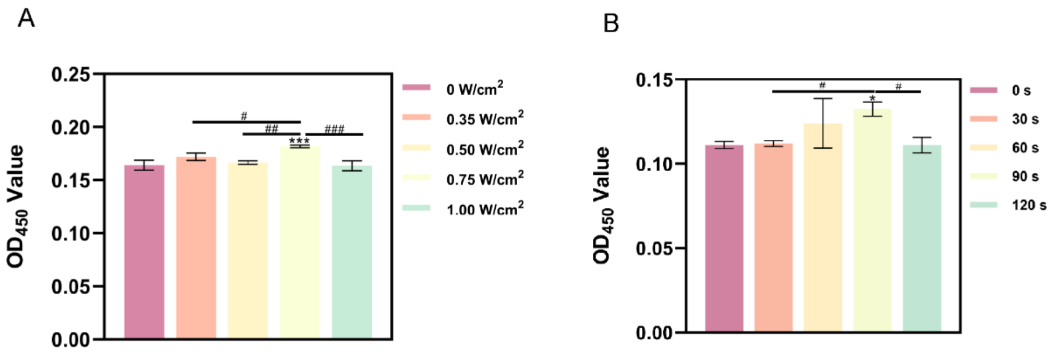

Supplementary Figure S2. Optimization of ultrasound parameters. (a-b) Cell proliferation under (a) different pulse strengths and (b) after different ultrasonic stimulation times. Values are expressed as mean ± standard deviation (n = 3). * p <0.05, ** p <0.01, *** p <0.001, and **** p <0.0001 compared with control group; $^{\#}$ p <0.05, $^{\#\#}$ p <0.01, and $^{\#\#\#}$ p <0.001 for comparison between groups.
OD: optical density.

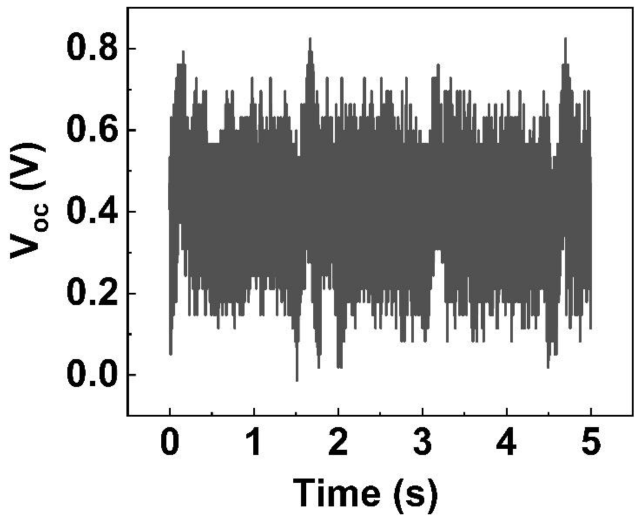

Supplementary Figure S3. Output voltage of the PHB/5%BT nanofiber scaffold with LIPUS stimulation in biological experiments. LIPUS: low-intensity pulsed ultrasound; PHB-BT: polyhydroxybutyrate-barium titanate.

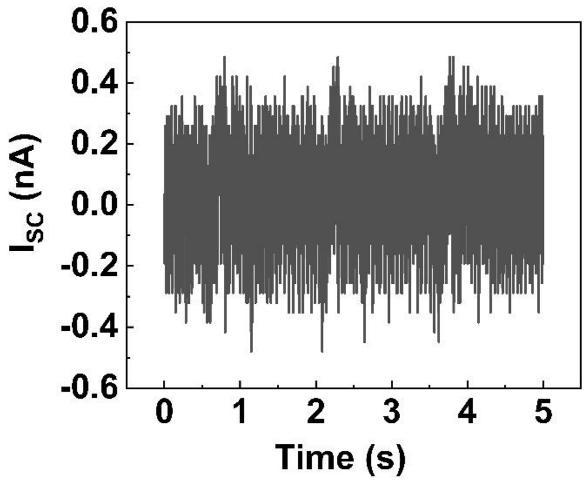

Supplementary Figure S4. Output current of the PHB/5%BT nanofiber scaffold with LIPUS stimulation in biological experiments. LIPUS: low-intensity pulsed ultrasound; PHB-BT: polyhydroxybutyrate-barium titanate.

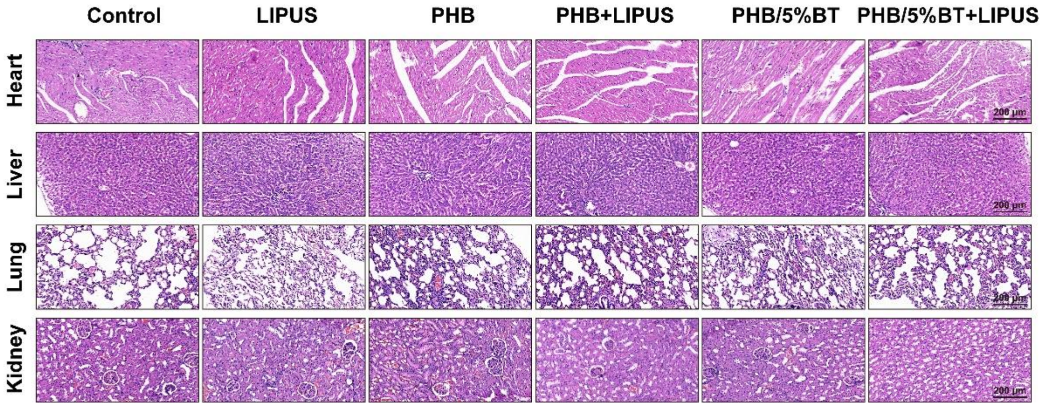

Supplementary Figure S5. Hematoxylin-eosin staining of rat heart, liver, lungs, and kidneys. LIPUS: low-intensity pulsed ultrasound; PHB-BT: polyhydroxybutyrate-barium titanate.

Table S1. Primers used for the qRT-PCR analysis

Gene name	Forward primer (5′–3′)	Reverse primer (5′–3′)
GADPH	TCCAGTATGACTCTACCCACG	CACGACATACTCAGCACCAG
RUNX2	GTGGCCAGGTTCAACGATCT	TGAGGAATGCGCCCTAAATCA
ALP	GTTACAAGGTGGTGGACGGT	ACAGTGGTCAAGGTTGGCTC
COl1a1	GATCCTGCCGATGTCGCTAT	GGGACTTCTTGAGGTTGCCA
OPN	CCAGCCAAGGACCAACTACA	CCAAGTGGCTACAGCATCTGA
OCN	GGCGCTACCTCAACAATGGA	GGCAACACATGCCCTAAACG
BMP2	TGCTCAGCTTCCATCACGAA	AATTTTGAGCTGGCTGTGGC
NFAT	AGCCCTACCATTTATTCTAC	CCCCTCTGTTACTTACCC
CaM	GGCATGGGACAATAACAA	TTACCGTCGGCATCTACT
CaSR	TTGCCCAGAACAAATCG	GAGCAGGCGTAGCTCACC
STIM1	TCTGAAGAGTCTACCGAAGC	CACATCACCATTGGCATC
CaN	ACAGTCAGGGGTTGTTCG	CCAGTTGTTTCCTTTTC
